# How low can we go with the dialysate flow? A retrospective study on the safety and adequacy of a water-saving dialysis prescription

**DOI:** 10.1093/ckj/sfae238

**Published:** 2024-08-08

**Authors:** Alicja Rydzewska-Rosołowska, Irena Głowińska, Katarzyna Kakareko, Adam Pietruczuk, Tomasz Hryszko

**Affiliations:** 2nd Department of Nephrology, Hypertension and Internal Medicine with Dialysis Unit, Medical University of Białystok, Białystok, Poland; 2nd Department of Nephrology, Hypertension and Internal Medicine with Dialysis Unit, Medical University of Białystok, Białystok, Poland; 2nd Department of Nephrology, Hypertension and Internal Medicine with Dialysis Unit, Medical University of Białystok, Białystok, Poland; Dialysis Center, Sokółka Poland; 2nd Department of Nephrology, Hypertension and Internal Medicine with Dialysis Unit, Medical University of Białystok, Białystok, Poland

**Keywords:** dialysate flow, dialysis adequacy, green nephrology, Kt/V, water

## Abstract

**Background:**

Green nephrology encompasses all initiatives in kidney care that have a positive impact on climate and environment. To prepare the dialysate, at least 120 L of water are needed for one 4-h session with a dialysate flow (Qd) set at 500 mL/min. A lower dialysate flow rate is associated with a significant reduction in the amount of water used. The aim of this study was to check whether change of Qd from 500 mL/min to 300 mL/min has a significant impact on dialysis adequacy.

**Methods:**

The study was a retrospective analysis. Due to administrative issues, a satellite dialysis center reduced their dialysate flow to 300 mL/min for a month. The center then increased Qd to 500 mL/min again. We analyzed laboratory data from 3 months before dialysate flow reduction, in the month with Qd reduced to 300 mL/min, and from 3 months thereafter with Qd set at 500 mL/min.

**Results:**

Twenty-four people were included in the final analysis. There were no significant changes in urea reduction ratio caused by lower rate of Qd [64.50 (61.75–71.00) vs 67.00 (63.00–72.25) vs 69.00 (63.75–72.25), analysis of variance F(2,46) = 0.71, *P *= .50]. Similarly, hemodialysis adequacy expressed by Kt/V did not differ at any Qd [1.23 (1.12–1.41) vs 1.25 (1.18–1.40) vs 1.35 (1.19–1.48), ANOVA F(2,46)* *=* *2.51, *P *= .09]. There was a small but statistically significant increase in mean predialysis potassium with lower Qd [potassium = 5.18 (95% confidence interval, 95% CI, 4.96–5.44) vs 5.46 (95% CI 5.23–5.69) vs 5.23 (95% CI 4.99–5.47) mmol/L at Qd = 500, 300 and 500 mL/min, respectively, *P *= .039].

**Conclusion:**

Reduction in dialysate flow rate to 300 mL/min seems safe and does not cause any short-term negative effects in this small study. Thus, we might be able to achieve a similar therapeutic effect while saving water consumption. Larger, long-term studies incorporating patient-reported outcome measures are needed to confirm the efficacy of this approach.

## Graphical Abstract

**Graphical Abstract:**
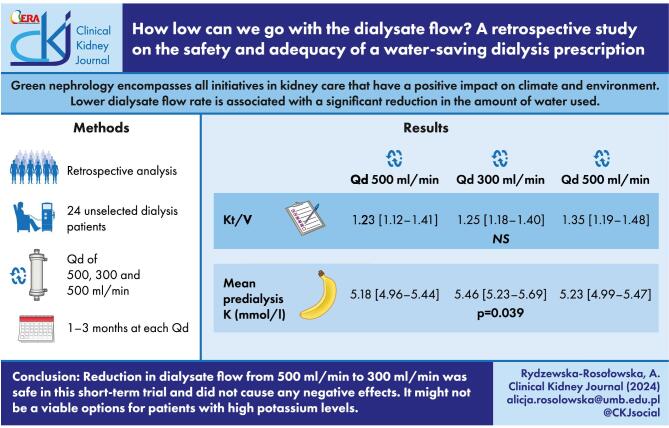

KEY LEARNING POINTS **What was known:**

- Lower dialysate flow (Qd) rate is associated with a significant reduction in the amount of water used for the hemodialysis procedure.
- Lowering the Qd might reduce the delivered single-pool Kt/V.
- There are scarce reports on whether Qd can be safely lowered during hemodialysis, and their results are mixed.

**This study adds:**

- There were no significant changes in urea reduction ratio caused by lower rate of Qd (lowered from 500 mL/min to 300 mL/min). Hemodialysis adequacy expressed by Kt/V did not differ at any Qd.
- Effectiveness of potassium removal during hemodialysis procedure expressed as a difference between serum potassium before and after HD was not significantly affected by decreased Qd. The mean increase of predialysis potassium evoked by decrease in Qd from 500 mL/min to 300 mL/min was 0.28 (95% confidence interval 0.05–0.5) mmol/L.
- Qd/Qb (blood flow) ratio decreased significantly without any negative impact on dialysis adequacy.

**Potential impact:**

- New ways are needed to make hemodialysis ecofriendly and to use water consciously. Minimizing dialysate flow might be one of them as shown in this study.
- Lowering the dialysate flow from 500 to 300 mL/min saves at least 48 L per dialysis session (when accounting only for the produced dialysate) and reduces the plastic waste burden generated by dialysate concentrate canisters.

## INTRODUCTION

Water is crucial for human health and survival and access to it has been recognized by the United Nations General Assembly as a human right [1]. Unfortunately, 27% of world population does not have access to safely managed drinking water [2]. With climate change being the biggest global health threat of the 21st century [3] the environmental impact of our everyday practices must be examined.

Nephrology is a resource-intensive specialty. Hemodialysis in particular consumes a lot of resources and has a high carbon footprint compared with other medical therapies [4, 5], while both hemodialysis and peritoneal dialysis produce a lot of consumable and disposal waste. These are the many reasons that prompted some nephrologists to promote “green nephrology,” or Blue Planet dialysis [6]. A global and inclusive initiative GREEN-K (Global Environmental Evolution in Nephrology and Kidney Care) with a mission to “promote and support environmentally sustainable and resilient kidney care globally through advocacy, education, and collaboration” was announced in 2023 [7].

Hemodialysis is certainly a lifesaving procedure but its impact on the environment is tremendous. A standard dialysis session lasts 4 h, and with a dialysate flow (Qd) of 500 mL/min requires 120 L of ultrapure water. Due to the inefficiency of reverse osmosis filtration, additional water is needed to prime, rinse and sterilize the system, all this might add up to as many as 500 L of water used for one hemodialysis session. All that is not used becomes waste (up to 380 L per session) and although some centers try to use this as so-called greywater (e.g. for cleaning and toilet flushing) [8, 9], new ways are needed to minimize water use.

A lower dialysate flow rate is associated with a significant reduction in the amount of water used for the hemodialysis procedure. Lowering the Qd has a potential negative impact however, as it might reduce the delivered single-pool Kt/V. Few reports trying to answer the question of whether dialysate flow can be safely reduced in hemodialysis patients have been published, and the results were mixed [10–13]. The aim of this study was to check whether change of dialysate flow from 500 mL/min to 300 mL/min has a significant impact on dialysis adequacy.

## MATERIALS AND METHODS

### Study design

During the COVID-19 surge in January 2022, due to administrative issues in a satellite dialysis center dialysate flow was reduced to 300 mL/min. The center increased the Qd back to 500 mL/min again in February 2022. We analyzed laboratory data from the 3 months before dialysate flow reduction (Qd = 500 mL/min, from October to December 2021), in the month with Qd reduced to 300 mL/min (January 2022) and in the 3 months thereafter with Qd set at 500 mL/min (from February to April 2022).

### Definitions

Urea reduction ratio (URR) was calculated based on pre and post dialysis urea values (taken from the arterial sampling port at the start of dialysis and 30 min post-dialysis session, respectively) and presented as a percentage. The formula used for calculation was: 100 ×  1 – (UreaPostHD/UreaPreHD).

Single-pool Kt/V was calculated using the Daugirdas formula [14].

Significant hyperkalemia was defined as serum potassium before hemodialysis above 6 mmol/L.

### Ethics

As the nature of the study was retrospective, a requirement for informed consent was waived. The study received approval of the Bioethical Committee of the Medical University of Białystok (number APK.002.30.2024).

### Statistical analysis

Arithmetic mean of analyzed data was calculated from the 3 months preceding and the 3 months following Qd decrease for statistical computations. Continuous variables are presented as a median together with the first and third quartiles (Q1–Q3). Categorical data are described as absolute and relative frequencies.

The null hypothesis assuming a lack of differences between three time points (Qd 500 mL/min, Qd 300 mL/min and Qd 500 mL/min) was checked by analysis of variance (ANOVA) with repeated measures calculation. The following tests were used to check whether the assumptions of ANOVA with repeated measures were met. The normality assumption was checked with the Shapiro–Wilk test for each time point. The sphericity assumption was assessed by computing Mauchly's test. Data were considered as extreme outliers if it was below Q1 – 3 × interquartile range (IQR) or above Q3 + 3 × IQR. If any assumption was violated, non-parametric Friedman test was used. Differences between categorical values were assessed with χ² test. The general linear model for correlated (repeated) measurements was used to calculate the mean differences and the corresponding 98.3% confidence intervals (CIs) in predialysis potassium level at three timepoints during the study duration.

As two patients died during the observation, their data were list-wise deleted before any statistical computation. Result robustness was tested in a sensitivity analysis, in which missing values were replaced with similar case imputation method.

A threshold of statistical significance was set at the *P*-value <.05. All statistical computations were done with R ver. 4.3.0 “Already Tomorrow.”

## RESULTS

### Study population

During the study duration all 26 patients dialyzed in satellite center were analyzed. Two of them died (in Month 6 and 7: one of the patients died due to heart failure and second due to stroke, both died in the hospital and admission potassium levels were 4.8 mmol/L and 4.3 mmol/L, respectively) during the observation period. Characteristics of the studied patients are presented in Table 1.

**Table 1:** Characteristics of studied population.

Parameter	Value
Demographics	
Age (years)	72.00 (64.75, 78.00)
Sex (males)	14 (58.30)
Dialysis vintage (months)	39.02 (23.96, 78.70)
Dry weight (kg)	69.50 (62.88, 84.25)
BMI (kg/m^2^)	26.81 (22.90, 28.98)
Comorbidities	
Diabetes mellitus	4 (16.70)
Heart failure	3 (12.50)
Laboratory	
Hemoglobin (g/dL)	11.00 (10.00, 11.25)
Phosphate	6.03 (5.06, 7.28)
Hemodialysis parameters	
Vascular access (catheter)	14 (58.30)
Dialyzer surface (m^2^)	1.80 (1.60–1.80)
Kt/V	1.23 (1.12, 1.41)
Blood flow rate (mL/min)	251.50 (249.75, 255.83)
ESA (IU/week)	7333.00 (4166.50, 11 250.00)

Data are presented as number (percentage) or median (IQR).

BMI: body mass index; ESA: erythropoiesis-stimulating agent.

### Parameters of hemodialysis procedure

As it was an observational study, first we examined whether other hemodialysis parameters potentially influencing on session effectiveness, apart from Qd, were constant during the observation period. The Friedman test revealed a small but significant difference in blood flow (Qb) rate across the three time points [251.50 (249.75–255.84) mL/min vs 254.00 (250.00–260.00) mL/min vs 251.34 (249.42–256.67) mL/min, Friedman's χ²(2) = 13.9, *P *= 0.001]. The Qd/Qb ratio decreased significantly from 1.99 (1.95–2.00) at Qd 500 mL/min to 1.18 (1.15–1.20) at Qd 300 mL/min and returned to 1.99 (1.95–2.00) after increasing Qd to 500 mL/min [Friedman's χ²(2) =35.6, *P *< .001]. There were no changes regarding the surface, type of dialyzers and time of hemodialysis session during the study duration. The temperature remained stable and was set at 37°C.

### Effect of decreased Qd on measures of hemodialysis adequacy

**Figure 1:**
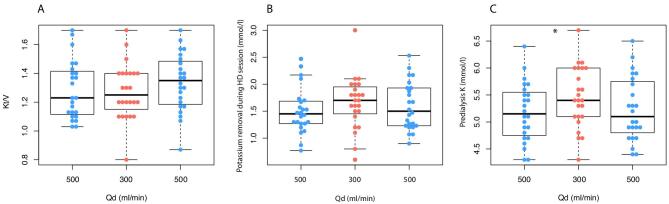
(**A**) Kt/V at different time points with different Qd. (**B**) Potassium removal rate at different time points with different Qd. (**C**) Predialysis potassium at different time-points with different Qd. ^*^Statistically significant difference.

There were no significant changes in URR caused by the lower rate of Qd [64.50 (61.75–71.00) vs 67.00 (63.00–72.25) vs 69.00 (63.75–72.25), ANOVA F(2,46) = 0.71, *P *= .50]. Similarly, hemodialysis adequacy expressed by Kt/V did not differ at any Qd [1.23 (1.12–1.41) vs 1.25 (1.18–1.40) vs 1.35 (1.19–1.48), ANOVA F(2,46) = 2.51, *P *= .09] (Fig. 1A).

### Effect of decreased Qd on potassium concentration

Effectiveness of potassium removal during hemodialysis procedure expressed as a difference between serum potassium before and after HD was not significantly affected by decreased Qd [1.45 (1.27–1.68) mmol/L vs 1.70 (1.48–1.93) mmol/L vs 1.50 (1.23–1.93) mmol/L, ANOVA F(2,46) = 1.58, *P *= .22] (Fig. 1B).

Mean predialysis potassium at Qd = 500 mL/min was 5.18 (95% CI 4.96–5.44) mmol/L, at Qd = 300 mL/min 5.46 (95% CI 5.23–5.69) mmol/L and at Qd = 500 mL/min 5.23 (95% CI 4.99–5.47) mmol/L [ANOVA F(2,46) = 3.81, *P *= .039] (Fig. 1C).

The mean increase of predialysis potassium evoked by decrease in Qd from 500 mL/min to 300 mL/min was 0.28 (95% CI 0.05–0.5) mmol/L. Follow-up increase in Qd from 300 mL/min to 500 mL/min resulted in mean predialysis potassium decrease –0.23 (95% CI –0.5 to 0.004).

Significant hyperkalemia was defined as serum potassium before hemodialysis above 6 mmol/L. Initially, during hemodialysis with Qd = 500 mL/min significant hyperkalemia was found in 17% of patients. The decrease of Qd to 300 mL/min did not evoke significant changes in the percentage of patients with serum potassium above 6 mmol/L, and it was equal to 12%. After increasing Qd up to 500 mL/min, 21% patients had a serum potassium value above 6 mmol/L (χ² = 0.6, *P *= .074).

### Effect of type of vascular access on the results

Patients dialyzed with permanent catheter had significantly lower Qb 250 (240–254) vs 255 (252–262) mL/min. Three-way ANOVA for repeated measures did not reveal any modifying effect of vascular access on predialysis potassium level [ANOVA F(1,22), *P *= .10], potassium removal [ANOVA F(1,22), *P *= .06] or Kt/V [ANOVA F(1,22), *P *= .57].

## DISCUSSION

Our study, performed in a real-life setting on the entire population of a small dialysis unit, suggests that reducing the dialysate blood flow during hemodialysis is safe and does not lower dialysis adequacy as measured by Kt/V, URR or potassium levels. On the other hand, it conveys important benefits, as theoretically lowering the dialysate flow from 500 to 300 mL/min saves at least 48 L per dialysis session (when accounting only for the produced dialysate) and reduces the plastic waste burden generated by dialysate concentrate canisters. In the studied dialysis unit (26 patients), assuming 156 dialysis sessions per patient/year it would mean a reduction in water use of 194 688 L per year. If we added to that calculation water of which less is rejected in the reverse osmosis process, less energy used during water purification process the magnitude of savings would be even greater.

Since the 1960s, the dialysate flow has been kept stable at 500 mL/min all around the world. During that time, however, we have changed the dialyzers that are used for our procedures, which may be one factor allowing the change of the standard prescription. On the other hand, it might have been possible before, as the first report of a patient dialyzed successfully for over a year with a dialysate flow of 100 mL/min comes from the group of Professor Belding Scribner [12].

The Qd/Qb ratio decreased significantly from 1.99 (1.95–2.00) at Qd 500 mL/min to 1.18 (1.15–1.20) at Qd 300 mL/min, and returned to 1.99 (1.95–2.00) after increasing Qd to 500 mL/min [Friedman's χ² = 35.6, *P *< .001]. Therefore, it seems that contrary to prior studies [11, 15] the flow ratio does not have to be fixed at 2:1 to achieve dialysis efficiency and that water can be saved without changes made to Qb.

The first larger study was published in 1981, on 20 carefully selected, stable dialysis patients being treated with a Qd of 300 mL/min for 24 months [16]. The authors reported no difference in weight, blood pressure, blood urea nitrogen, hematocrit, creatinine, bicarbonate or potassium, and no adverse symptoms. The Kt/V was not analyzed due to the fact that it was developed by Gotch and Sargent in 1985 [17], so it is unknown whether modern measures of dialysis adequacy were attained in the study.

The next study was done on 23 hemodialysis patients and involved changing the dialysis prescription from Qd of 300 mL/min to 500 mL/min and finally 800 mL/min for at least 3 weeks [10]. In contrast to our study and the few others reported, it was the only negative study. The authors did see a meaningful difference in Kt/V with different dialysate flow rates. They reported the proportion of patients not achieving adequacy and it was 56%, 30% and 13%, respectively, for greater dialysate flow rates. We are unable to fully explain why this study reported different results, although the percentage of patients not reaching the desired Kt/V was large and the authors attribute it to the “individualized dialysis prescription” in their center which takes into account residual renal function.

The only modern long-term study (6 years of observation, 71 patients) of Qd reduction—400 mL/min versus 500 mL/min—comes from Colombia and has been reported as an abstract only [18]. The positive outcome of the study must be therefore interpreted with caution and cannot be fully considered until a peer-reviewed publication is made available.

The most recent study on the safety of reducing dialysate flow in the dialysis prescription is the one performed by Solomon *et al*. [13]. The study population was similar to ours—they analyzed 30 patients, also dialyzed for 1 month but with three different hemodialysis prescriptions: Qd of 500 mL/min and a temperature of 37°C, Qd of 500 mL/min and a temperature of 38.5°C, and Qd of 300 mL/min and a temperature of 38.5°C. They reported no significant difference in the achieved mean URR or single-pool Kt/V between the compared prescriptions. Unfortunately, due to the concerns of potential hemodynamic instability with higher temperatures patients with an ejection fraction (EF) of <40%, recurrent intradialytic hypotension and autonomic neuropathy were excluded. The study, unlike ours, did not include a group dialyzed with a Qd of 300 mL/min and a standard temperature. They did not report any patient discomfort due to the high temperature, but did witness an episode of symptomatic hypotension resulting in dialysis termination. They did not report on potassium levels in the studied patients, and we well know that hypokalemia is associated with hypothermia [19], and the converse might be also true (higher potassium levels at higher temperature) as temperature-dependent K+ channels probably play a role in the observed potassium changes [20]. We, on the other hand, report on potassium removal during the study period. The finding that the mean increase of predialysis potassium evoked by decrease in Qd from 500 mL/min to 300 mL/min was 0.28 (95% CI 0.05–0.5) mmol/L, which suggests that decreasing blood flow might not be appropriate in patients with high predialysis potassium levels.

Our study is not without several limitations. First of all, it was retrospective in nature and the number of studied patients is small. The population might not representative (probably due to small numbers)—e.g. only 17% had diabetes. Dialysate flow was changed only for a period of 1 month, so it is hard to extrapolate on what would be the long-term consequences of such dialysis prescription. The follow-up period is also short. We unfortunately (due to the retrospective nature of the study) were not able to check any patient-reported outcome measures. Nevertheless, it proves that such trials probably can be done safely, and should be done if we want to minimize water usage and plastic waste generation. On the other hand, the main strength of our study is that no patients in the center were excluded due to e.g. age, low EF, recurrent symptomatic hypotension or other reasons, and we changed only one parameter—the dialysate flow—with other prescribed factors (especially temperature) being identical which makes the interpretation easier. It suggests that at least in the short-term a Qd of 300 mL/min does not cause significant changes in URR or Kt/V and might be safe in patients without high predialysis potassium levels.

Whether green, blue or gray, new ways are needed to make hemodialysis ecofriendly and to use water consciously. Minimizing the dialysate flow might be one of them, as shown in this small study. Larger, long-term, randomized studies are needed to determine the safety and efficacy of this approach.

## Data Availability

The data underlying this article will be shared on reasonable request to the corresponding author.
